# Ocular parameters of biological ageing in HIV-infected individuals in South Africa: Relationship with chronological age and systemic biomarkers of ageing^☆^

**DOI:** 10.1016/j.mad.2013.08.002

**Published:** 2013-09

**Authors:** Sophia Pathai, Paul G. Shiels, Helen A. Weiss, Clare E. Gilbert, Tunde Peto, Linda-Gail Bekker, Robin Wood, Tien Y. Wong, Stephen D. Lawn

**Affiliations:** a International Centre for Eye Health, Department of Clinical Research, Faculty of Infectious and Tropical Diseases, London School of Hygiene & Tropical Medicine (LSHTM), Keppel Street, London WC1E 7HT, UK; b Desmond Tutu HIV Centre, Institute of Infectious Diseases and Molecular Medicine, Faculty of Health Sciences, University of Cape Town, Anzio Road, Observatory 7925, Cape Town, South Africa; c Institute of Cancer Sciences, College of Medical, Veterinary & Life Sciences, University of Glasgow, Glasgow, UK; d MRC Tropical Epidemiology Group, Faculty of Epidemiology and Population Health, LSHTM, Keppel Street, London WC1E 7HT, UK; e NIHR Biomedical Research Centre for Ophthalmology, Moorfields Eye Hospital NHS Foundation Trust and UCL Institute of Ophthalmology, 162 City Road, London EC1V 2PD, UK; f Singapore Eye Research Institute, National University of Singapore, 11 Third Hospital Avenue, Singapore 168751, Singapore; g Department of Clinical Research, Faculty of Infectious and Tropical Diseases, LSHTM, Keppel Street, London WC1E 7HT, UK

**Keywords:** Telomeres, CDKN2A, Lens density, Retinal vessel calibre, HIV

## Abstract

HIV-infected individuals have an increased risk of age-related morbidity despite antiretroviral treatment (ART). Several anatomic and functional ophthalmological parameters are associated with increasing chronological age. These may, therefore, potentially serve as biomarkers of ageing. We investigated associations between ocular parameters (lens density, retinal vessel calibre, corneal endothelium and retinal nerve fibre layer thickness) and two ‘cellular’ biomarkers of ageing (leukocyte telomere length and CDKN2A expression) and with frailty in a cross-sectional study of 216 HIV-infected individuals. All ocular parameters, telomere length and frailty were associated with chronological age, whereas CDKN2A expression was not. Retinal venular calibre and lens density were associated with shorter telomere length (*p*-trend = 0.04, and 0.08, respectively), whereas CDKN2A expression and frailty status were not associated with ocular parameters. Longitudinal studies are warranted to assess the integration of retinal vascular calibre and lens density with systemic markers to develop an overall index of biological ageing in HIV infection.

## Highlights

• HIV is associated with age-related morbidity despite antiretroviral treatment.
• Ocular age-related parameters may serve as biomarkers of ageing.
• Lens density may have a role in the determination of biological age in HIV infection.

## 1 Introduction

Major reductions in HIV-associated mortality have occurred largely due to the global scale-up of antiretroviral therapy (ART). However, evidence is emerging that patients receiving ART are at an increased risk of age-related non-AIDS morbidity and mortality compared with HIV-seronegative individuals (Deeks, 2009; Guaraldi et al., 2011; Lohse et al., 2007). Several of these conditions are classically associated with the normal ageing process but appear to occur at an earlier age in HIV-infected persons compared to age-matched HIV-seronegative individuals. It is possible that not only are HIV cohorts ageing chronologically, but they may also be undergoing accelerated biological ageing.

Chronological age is an imprecise measure of biological ageing, due to inter-individual differences in rates of ageing. The disconnection between chronological age and lifespan has led to a search for effective and validated biomarkers of ageing (BoA), defined as “biological parameters of an organism that either alone or in some multivariate composite will better predict functional capability at some late age than will chronological age”(Baker and Sprott, 1988). In HIV infection, heightened inflammation and immune dysfunction are likely to play a role in accelerated ageing (Deeks, 2009). This has stimulated epidemiological assessment of several biomarkers of inflammation and immune dysfunction, including C-reactive protein (CRP), interleukin-6 (IL-6) and D-dimer and their associations with age-related morbidity and HIV infection (Nixon and Landay, 2010).

Only two validated BoA, telomere length (TL) and CDKN2A expression, have so far been found to satisfy the majority of the criteria proposed by Baker and Sprott (1988). Telomeres are nucleoprotein complexes at the ends of eukaryotic chromosomes. Their DNA component shortens with somatic cell division and upon reaching a critically short length, a DNA damage signal leads to growth cycle arrest, resulting in replicative senescence (Saretzki and Von Zglinicki, 2002; von Zglinicki, 2002). Telomere shortening is associated with increasing chronological age and several pathologies. Telomere length (TL) may be useful as a composite measure of healthy ageing, but not as a BoA when used in isolation (Der et al., 2012; von Zglinicki, 2012). Expression levels of the cell cycle regulator CDKN2A may represent a more robust BoA in healthy ageing (Shiels, 2010). CDKN2A acts as a tumour suppressor and maintains cells in a state of growth arrest, both in replicative and stress induced-senescence. Increasing levels of CDKN2A transcriptional expression occur with increasing age and decreasing function of solid organs and peripheral blood leucocytes (PBLs) (Koppelstaetter et al., 2008; Krishnamurthy et al., 2004; Liu et al., 2009; McGlynn et al., 2009). However, in disease or pro-inflammatory states such as HIV infection, evaluation of CDKN2A expression may be compromised by mechanisms such as stress-induced premature senescence (SIPS) (Shay and Wright, 2000). Furthermore, measurement of telomere length may be affected by the use of nucleoside reverse transcriptase inhibitors (NRTIs) (Yamaguchi et al., 2001). Thus, novel biomarkers are required to evaluate biological ageing in HIV infection (Lundgren et al., 2010).

Certain anatomic and functional parameters of the eye change with increasing chronological age. They may, therefore, serve as potential biomarkers of ageing (Pathai et al., 2013e). We have reported on ocular parameters related to the accelerated ageing phenotype in HIV, including retinal vessel calibre, objective measurement of lens density and assessment of the corneal endothelium (Pathai et al., 2012, 2013c,d). Furthermore, we have shown frailty to be part of this phenotype (Pathai et al., 2013a). Frailty is a functional state characterised by an increased risk of multiple pathologies, low physical activity and slow motor performance (Fried et al., 2001). Frailty predicts cognitive and physical decline and is associated with an increased risk of morbidity and mortality, and may therefore act as a ‘clinical’ biomarker of ageing (Fried et al., 2001). There are limited data on how these parameters correlate with cellular BoA in the context of HIV as such markers are typically used in the evaluation of healthy biological ageing. In addition, there are few data relating to biological ageing in sub-Saharan Africa, a region where the population of HIV-infected elderly people is rapidly expanding and where the AIDS epidemic is most severe (Mills et al., 2011).

With the need for novel BoA that could be valid in disease as well as health, the aim of this study was to investigate the association between eye parameters, frailty and cellular BoA in HIV infection.

## 2 Methods

### 2.1 Study population

HIV-infected individuals aged ≥ 30 years from a community HIV treatment clinic in a township of Cape Town, South Africa (Hannan Crusaid Centre, Gugulethu) were recruited as HIV-infected ‘cases’ as part of a case-control study investigating HIV and ageing (Pathai et al., 2012, 2013a). Participants in this study were receiving ART at the time of enrolment. Socio-demographic information and medical history were obtained by interviewing participants in their first language (Xhosa or English). Data collected included factors known to affect ageing (e.g. UV exposure, smoking history). All participants underwent a full ophthalmic examination including measurement of visual acuity, evaluation by slit lamp microscopy and indirect ophthalmoscopy.

The study was approved by the Ethics Committees of the London School of Hygiene and Tropical Medicine and the University of Cape Town Faculty of Health Sciences, and adhered to the tenets of the Declaration of Helsinki. Written informed consent was obtained from all participants.

### 2.2 Anthropometry, blood pressure and physical function including frailty assessment

Blood pressure (BP) was measured with a digital sphygmomanometer. Mean arterial blood pressure (MABP) was defined as two-thirds of the diastolic plus one-third of the systolic BP (Wong et al., 2003). Hypertension was defined as a systolic BP of 140 mmHg or higher, diastolic BP of 90 mmHg or higher, or the combination of self-reported high BP diagnosis and the use of anti-hypertensive medications (Wong et al., 2005). Body mass index (BMI) was defined as weight (in kilograms)/height^2^.

Physical frailty was defined by the presence of ≥3 of 5 criteria: (i) unintentional weight loss (self reported and verified from clinic records where possible) (ii) self-reported low physical activity, (iii) self-reported exhaustion, (iv) weak grip strength and (v) slow walking time. Pre-frailty was defined as the presence of one or two of these criteria. Detailed information is available in the Supplementary Methods.

## 3 Blood-based biomarkers

### 3.1 DNA/RNA extraction

DNA was extracted from PBLs using the Maxwell™ Automated Purification System according to manufacturer's instructions (Promega, USA). DNA concentration and purity were quantified by Nanodrop Spectrophotometer (ThermoFisher Scientific, USA). RNA was extracted using Trizol reagent (Invitrogen, UK) following manufacturer's guidelines. DNA/RNA extraction was performed in Cape Town and samples shipped on dry ice to the University of Glasgow.

#### 3.1.1 Telomere length determination

TL were determined by QPCR following the method of Cawthon (2002). TL determination was performed blindly using a Roche Light Cycler LC480. Briefly, telomere length analyses were performed in triplicate for each sample, using a single-copy gene amplicon primer set (acidic ribosomal phosphoprotein, 36B4) and a telomere-specific amplicon primer set (Koppelstaetter et al., 2008). Refer to Supplementary Methods for further detail.

#### 3.1.2 CDKN2A expression determination

Relative quantitative real-time PCR (qRT-PCR) was used to estimate mRNA levels corresponding to the candidate senescence associated gene – CDKN2A. Expression levels were measured against a reference hypoxanthine phosphoribosyltransferase (HPRT) housekeeping gene on an ABI Prism(R) 7500 Sequence Detection System. Sequences of human TaqMan™ Primer/Probe sets were designed by Primer Express algorithm (Applied Biosystems, Austin, TX, USA). The comparative threshold cycle method (ΔΔ*CT*)(Livak and Schmittgen, 2001) was employed to quantify relative gene expression.

## 4 Ocular biomarkers

The following four ocular parameters were selected (Table 1). Detailed methods are supplied in the Supplementary Methods.

(i) Lens density: Pentacam imaging (Oculus, Wetzlar, Germany) was used to obtain “Scheimpflug images” of the lens and an objective estimate of lens density on a continuous scale. Lens density increases with increasing chronological age.
(ii) Retinal vessel calibre: Participants had stereoscopic 30° colour retinal photographs taken under pharmacological pupil dilation with a fundus camera (CF-2; Canon Inc., Tokyo, Japan). Vessel calibre indices were determined in a semi-automated manner using the IVAN computer program (Singapore Eye Research Institute, Singapore) and a standardized protocol described previously (Wong et al., 2004). Narrowing of retinal arterioles is associated with increasing chronological age (Leung et al., 2003; Wong et al., 2003).
(iii) Corneal endothelial cell parameters: A non-contact specular microscope was used (SP02, CSO, Florence, Italy). The operator focused and aligned a real-time image of the participant's eye. Endothelial cell parameters were automatically calculated from this image by the microscope software. Endothelial cell density (ECD) decreases with age, whereas the change in cell size (coefficient of variation) increases with age. The proportion of cells with six sides (hexagonality index) decreases with age.
(iv) Retinal nerve fibre layer (RNFL): measured using Spectral OCT/SLO optical coherence tomography (Opko/OTI Inc., Miami, FL) which uses a scanning laser diode of 830 nm to provide images of ocular microstructures. A peri-papillary (around optic nerve head) protocol inbuilt in the software was used to determine the average and quadrant-specific RNFL thickness (superior, inferior, temporal and nasal). The RNFL becomes thinner with increasing age (Chi et al., 1995; Kanamori et al., 2003).

## 5 Statistical analysis

One eye was randomly selected for analysis. Where an eye was not available, for example due to trauma or corneal opacity, the contralateral eye was used. Analyses were performed using Stata 12 (Stata Corp., College Station, TX). Clinical and biological data were summarized as the median with interquartile range (IQR) or mean with standard error (SE), as appropriate. Analyses were conducted on log10-transformed values of telomere length and mean CDKN2A expression to satisfy the assumption of normally distributed residuals. Results are displayed back-transformed to the original scale. Validation of the biomarkers was performed using linear regression models with age in years as a continuous or categorical variable. Ocular biomarker measurements were divided into quartiles. Univariable linear regression was performed to compare the quartiles of ocular parameters with mean telomere length and CDKN2A expression and frailty status respectively. Multivariable linear regression models were used to examine the relationships of telomere length, CDKN2A expression as the respective dependent variable with ocular biomarker quartiles and frailty status and explanatory variables (age group [30–39; 40–49; >50 years], gender, MABP; BMI, smoking, UV exposure, current and nadir CD4 count, peak and current HIV viral load) as independent variables. Marginal adjusted means for telomere length and CDKN2A expression were estimated at the mean value of covariates in the model. The Wald test was used to assess statistical significance of the association of each ocular parameter on systemic biomarker levels.

## 6 Results

### 6.1 Participant characteristics and biomarker distributions

216 participants underwent assessment. The median age was 40 years (IQR: 35–46) and 25% (*n* = 54) were male. Characteristics of the participants by gender are given in Supplementary Table S1. Women reported less alcohol consumption and cigarette use compared to men (*p* < 0.0001 for both). Men had lower BMI and lower current CD4 counts (*p* < 0.0001 and *p* = 0.04, respectively). The number of participants providing data for each parameter varied, as not every participant was able to complete all ophthalmic tests or had a blood sample available for analysis. Summary statistics for each biomarker, stratified by gender and age group are displayed in Supplementary Table S2. For the majority of biomarkers, there was no evidence of gender differences; however for endothelial cell parameters, the cell density was greater in women compared to men, and women were more frail than men (*p* = 0.03 for both).

### 6.2 Validation of biomarkers against chronological age

All biomarkers were initially validated against chronological age in a HIV-seronegative control group of similar age and gender, recruited as part of a case-control study (Pathai et al., 2013b). Assessment within this HIV-infected study population was subsequently performed (Supplementary Table S3). Telomere length was associated with chronological age (*p* = 0.03), however CDKN2A expression was not (*p* = 0.25). All ocular parameters except the RNFL nasal quadrant and hexagonality index of endothelial cells were associated with chronological age, therefore these ocular parameters were not analysed further. The association of the prospective biomarkers with chronological age is presented in Supplementary Figures 1a–o. The *R*-squared values of the regressions against chronological age were highest for lens density parameters (linear lens density *R*^2^ = 0.63). All analyses thereafter were adjusted for age, gender and other possible confounding variables related to the parameter of interest.

### 6.3 Association of ocular parameters with blood-based biomarkers

Shorter telomere length was associated with increasing retinal venular diameter (*p*-trend = 0.04) – Table 2. Telomere length was not related to retinal arteriolar diameter. Table 3 reports associations with lens density: there was a trend of shorter telomere length with increasing lens density (*p*-trend = 0.08). Endothelial cell parameters and RNFL thickness were not related to TL or CDKN2A expression (data not shown).

### 6.4 Association of frailty status with blood-based and ocular biomarkers

There was no association of frailty status with either telomere length or CDKN2A expression (*p* = 0.54 and *p* = 0.76, respectively). Similarly, none of the ocular parameters were associated with frailty (data not shown).

## 7 Discussion

We compared several ocular parameters and frailty, a clinical correlate of ageing, with TL and CDKN2A expression as established and validated biomarkers of ageing. Retinal venular diameter was the most informative ocular biomarker, showing increased venular diameter with decreasing TL. Objective measurement of lens density also showed a strong trend of decreasing TL with increasing lens density. In contrast, CDKN2A expression, a validated biomarker of ageing in health, was not associated with frailty or any of the ocular parameters. These findings suggest that the ocular lens and retinal vasculature, which reflect different physiological systems, may have a role to play in the determination of biological age and ageing trajectories in HIV infection, which may differ from that seen in physiological ‘healthy ageing’.

We have already established in this same study population that HIV infection is associated with increased frailty (Pathai et al., 2013a), changes in retinal vessel calibre, lens density and in corneal endothelial cells that are consistent with accelerated ageing (Pathai et al., 2012, 2013c,d). These findings of an HIV-related accelerated ageing phenotype, suggest there will be important clinical and health systems implications as HIV-infected populations continue to increase and live longer. Associations of these ocular and clinical parameters with validated BoA have not been investigated to date. The need for evaluated biomarkers in determining biological age is gaining increasing importance as data emerge about the excess risk of age-related morbidity in HIV-infected individuals despite suppression of viral load (Lundgren et al., 2010).

Of a wide range of BoA evaluated, only TL and CDKN2A are thought to meet the Baker and Sprott criteria (Baker and Sprott, 1988). The majority of putative BoA assessed fail on the basis that they are unable to predict functional capacity in the absence of disease (Simm et al., 2008b). The issue is further compounded in HIV infection as BoA are typically used to evaluate physiological, ‘healthy’ ageing. Measurement of ‘accelerated’ biological ageing HIV infection may involve different mechanisms, and therefore render some BoA invalid in their measurement. This is pertinent to the lack of association between CDKN2A and chronological age in our HIV-infected study population, where the disease state decouples CDKN2A expression from correlation with chronological ageing. We have found CDKN2A expression to be significantly higher in HIV-infected individuals compared to age- and gender-matched controls (Pathai et al., unpublished data). However, as well as functioning as a tumour suppressor, CDKN2A is also a component of stress-induced premature senescence (SIPS) (Shay and Wright, 2000) which prevents T-cell replication following acute insult (Liu et al., 2011). The decoupling of the relationship between CDKN2A expression and chronological age in HIV infected individuals is a direct consequence of HIV-associated premature T-cell senescence (Deeks, 2011), and lack of further T cell replication with HIV infection. SIPS causes populations of cells to ‘freeze’ in time with respect to age, confounding the determination of rate changes in replicative senescence and therefore biological ageing. The Baker and Sprott criteria state “The biomarker should reflect some basic biological process of ageing and certainly not the predisposition toward a disease state or some error in metabolism” (Baker and Sprott, 1988). Thus, the induction of SIPS by CDKN2A in disease states such as HIV infection suggests that this is an unreliable BoA in these circumstances.

Telomere length has also been shown to be significantly shorter in HIV-infected individuals compared to HIV-seronegative controls in this population (Pathai et al., unpublished data), and may better satisfy the Baker and Sprott criteria in disease states. However, telomere attrition is affected by psychosocial confounders, genetics and potentially by NRTIs used in the treatment of HIV infection (Yamaguchi et al., 2001), leading to the suggestion TL may be useful as a composite measure of healthy ageing, but not as a BoA when used in isolation (Der et al., 2012; von Zglinicki, 2012). The trend of increasing lens density with shortening of telomere length is intuitive, and the strong association of lens density with chronological age (when compared to TL), suggests that in this population, lens density may be an attractive BoA as it is less susceptible to changes caused by systemic disease states. Logistical constraints precluded assessment of telomere length in T-cell subsets, which has been advocated in the context of HIV infection (Aviv et al., 2006). While we acknowledge that this would give a higher resolution data set, the primary aim of this study was to provide preliminary data to inform future work and assessment of telomere length in PBLs has been the approach used by others in this field (Leeansyah et al., 2013; Malan-Muller et al., 2013). It is also important to consider the role of ART, particularly NRTIs, in the context of accelerating mitochondrial ageing (Smith et al., 2012). For example, NRTIs may accelerate aspects of intrinsic biological ageing via clonal expansion of mtDNA mutations (Payne et al., 2011). HIV and/or HAART exposure are also associated with increased prevalence of AC/TG mtDNA mutations in mothers and show a similar tendency in infants exposed during pregnancy (Jitratkosol et al., 2012). This suggests that mtDNA mutations may affect the ageing trajectories of HIV and ART-exposed populations.

The frailty phenotype was initially described in HIV-infected individuals in 2007. In this study the prevalence of frailty among 55-year-old men infected with HIV for ≤4 years was similar to that of uninfected men ≤ 65 years old (Desquilbet et al., 2007). Similarly, we have found an increased prevalence of frailty in this study population (Pathai et al., 2013a). However, we did not detect an association between frailty and cellular BoA or ocular parameters. In contrast, in an HIV-seronegative study population from the same community we found increasing lens density to be associated with frailty status (Pathai et al., unpublished data). This suggests that the frailty phenotype in HIV, while sharing some characteristics with the original description in the geriatric population (Fried et al., 2001), has other characteristics perhaps unique to HIV infection and/or use of ART. This is corroborated by the finding that CD4 count is also associated with the development of frailty (Desquilbet et al., 2009; Pathai et al., 2013a), suggesting that compromise of the immune system in HIV-infected individuals contributes to the development of the frailty phenotype. Furthermore, HIV-infected frail individuals have higher concentrations of pro-inflammatory cytokines such as IL-6, and tumour necrosis factor-alpha (TNF-α), and C-reactive protein (Margolick et al., 2012). Age-related comorbidities and AIDS predict conversion from being non-frail to frail, and having a persistent frailty-like phenotype before ART initiation also predicts mortality (Desquilbet et al., 2011). Collectively, these findings suggest that frailty in HIV infection is not simply an accelerated version of geriatric frailty, and so the lack of relationship with BoA at a cellular or ocular level may be expected.

Retinal arteriolar and venular calibre typically narrow with increasing chronological age (Leung et al., 2003; Wong et al., 2003), thus shortening of TL might be expected to be associated with narrowing of venular diameter. However, our observation that shortened TL was associated with increasing retinal venular diameter is nevertheless plausible in the context of HIV infection. Inflammation is recognised as a key pathogenic process in HIV infection, and also in HIV-related accelerated ageing (Deeks, 2009). Larger retinal venular calibre is associated with systemic inflammatory markers such as CRP, fibrinogen, IL-6 and smoking, independently of age (Ikram et al., 2004; Klein et al., 2006; Wong et al., 2006). This suggests that the heightened inflammatory processes observed in HIV infection are consistent with a biologically aged phenotype as manifest by shorter TL. Patients with HIV infection are at increased risk of cardiovascular disease (Triant, 2012). Although factors potentially contributing to this elevated risk include traditional cardiovascular risk factors and antiretroviral medications, it is likely that inflammatory and immunologic factors also contribute. Vascular endothelial dysfunction is also associated with larger retinal venules independent of traditional cardiovascular risk factors (Nguyen et al., 2010). We therefore postulate that HIV-related inflammation and/or endothelial dysfunction manifests as retinal venular dilation and contributes to the biologically aged phenotype. Furthermore, retinal arteriolar narrowing which is associated with increased cardiovascular risk (Wong et al., 2002), is related to increasing duration of ART, independent of age (Pathai et al., 2012). Thus, assessment of retinal vessel diameters may be a novel and non-invasive method of assessing vascular risk and biological ageing in this population.

This study has some limitations. The gender composition of participants was three-quarters female, however this is reflective of the HIV epidemic in Africa. There were also differences between genders (smoking, alcohol consumption) that may truly exist or may have been misclassified (e.g. misreporting true smoking habits) and this could have confounded associations of the ocular parameters with the other biomarkers. Participants were recruited from a community of considerable socio-economic deprivation, and therefore likely to have been exposed to factors known to increase biological ageing such as high UV exposure from outdoor work. Therefore, our data might over-estimate associations related to ageing. Lastly, as study participants are of African ancestry, our results are generalizable to the African population.

Biomarkers are used extensively in the assessment of HIV infection, from monitoring of CD4 count and HIV RNA viral load to assess response to ART, to the use of indices such as the (Veterans Aging Cohort Study) VACS Index to predict morbidity and mortality (Justice et al., 2012). Candidate biomarkers of ageing have been proposed in HIV-uninfected elderly population cohorts in other settings, as well as their relationship with frailty e.g. the Newcastle 85+ study (Collerton et al., 2012; Martin-Ruiz et al., 2011). Longitudinal studies in different populations are needed to assess how ocular parameters change over time in relation to blood-based biomarkers and to other candidate biomarkers that have been previously evaluated (Martin-Ruiz et al., 2011; Simm et al., 2008a). Our findings indicate that measurement of biological ageing in HIV infection may be complicated by inflammation and immune dysfunction such that BoA validated in healthy ageing are no longer accurate predictors. This suggests that other biomarkers are needed; our data indicate that measurement of lens density and retinal vessel calibre may be useful additions in the assessment of biological ageing. We have proposed a research agenda to further define and validate ocular biomarkers of ageing (Pathai et al., 2013e). Vessel calibre assessment may provide information about inflammation and systemic vascular changes, whereas lens density evaluation may be indicative of true biological ageing. Longitudinal studies are warranted to assess the integration of these data with systemic markers to develop an overall estimate of biological ageing and possibly mortality risk. In conclusion, the eye may prove to be a useful addition in the assessment of biological ageing and to improve our understanding of HIV-related accelerated ageing.

## Funding

This work was supported by a Wellcome Trust grant awarded to S.P. (grant number: 090354/Z/09/Z). S.D.L. is funded by the Wellcome Trust (grant number 088590). T.P. is funded by NIHR BRC at MEH and IoO.

## Figures and Tables

**Table 1:** Biomarkers of aging, methods of measurement and the impact of aging.

Anatomical site	Parameter	Method of measurement	Age-related changes
*Peripheral blood leukocytes*	Telomere length (TL)	qPCR	TL shortens
	CDKN2A expression	qRT-PCR to estimate mRNA levels	Increased expression

*Corneal endothelium*	Endothelial cell density (ECD)	Specular microscopy	Decreased ECD
	Coefficient of variation (CV)		Increased CV
	Hexagonality index (Ex)		Decreased Ex

*Lens*	Lens opacity	Pentacam-lens densitometry	
	Linear value		
	Peak		All increase
	3-D average		

*Retina*	Retinal nerve fibre layer (RNFL) thickness	Optical coherence tomography (OCT)	Thinner RNFL – all quadrants
	(Average, superior, inferior, nasal, temporal quadrants)		
		Semi-automated retinal analysis software applied to fundus photographs	Reduced diameter of arterioles and arterio-venous ratio (AVR)
	Retinal vessel caliber		

*Systemic*	Frailty status		
	Non-frail (no criteria)	Assessment of walking speed, grip strength, self-report of weight loss, exhaustion and low physical activity	
	Pre-frail (1–2 criteria)		Frailty status increases
	Frail: ≥3 of 5 criteria		

**Table 2:** Association of retinal arteriolar and venular diameter with systemic biomarkers.

Vessel parameter	N	TL	P	N	CDKN2A	P
		Rel T/S			Mean	
*Retinal arteriolar quartiles (μm)*						
1st	38	0.89		37	0.37	
(113.16–150.13)		(0.81–0.97)			(0.29–0.47)	
2nd	42	0.87		40	0.54	
(150.56–161.06)		(0.80–0.95)			(0.43–0.68)	
3rd	62	0.92		61	0.43	
(161.53–171.89)		(0.87–0.99)			(0.36–0.51)	
4th	54	0.93	*p*-trend	53	0.44	
(172.79–207.80)		(0.87–1.00)	0.25		(0.36–0.54)	0.19

*Retinal venular quartiles (μm)*						
1st	66	1.04		64	0.54	
(218.31–258.35)		(0.92–1.17)			(0.39–0.74)	
2nd	57	0.89		56	0.41	
(258.78–269.99)		(0.83–0.95)			(0.34–0.49)	
3rd	40	0.87		40	0.48	
(270.24–281.54)		(0.79–0.97)			(0.36–0.63)	
4th	33	0.74	*p*-trend	31	0.31	
(281.7–326.08)		(0.63–0.86)	0.04		(0.20–0.51)	0.15

Adjusted for age, gender, smoking, BMI, hypertension and venular/arteriolar retinal calibre (where appropriate) and HIV-related co-variates (current and nadir CD4 count, current and peak viral load, ART duration and ART type).

**Table 3:** Association of lens density with systemic biomarkers, *n* = 198.

Lens density^a^	N	TL	P	N	CDKN2A	P
		Rel T/S			Mean	
*Linear quartiles*						
1st	48	0.95		47	0.42	
(7.5–9.2)		(0.87–1.03)			(0.33–0.55)	
2nd	54	0.97		53	0.45	
(9.25–9.8)		(0.90–1.04)			(0.37–0.55)	
3rd	48	0.87		48	0.44	
(9.85–10.7)		(0.81–0.94)			(0.36–0.55)	
4th	48	0.84	*p*-trend	46	0.48	0.94
(10.8–13.9)		(0.77–0.93)	0.08		(0.36–0.62)	

Adjusted for age, gender, smoking, UV exposure and HIV-related parameters (current and nadir CD4 count, current and peak viral load, ART duration and ART type).

a Measured on a continuous scale 0–100, 100 being an opaque (completely dense) lens 4th quartile denotes aged phenotype.

## References

[bib0005] Aviv A., Valdes A.M., Spector T.D. (2006). Human telomere biology: pitfalls of moving from the laboratory to epidemiology. Int. J. Epidemiol..

[bib0010] Baker G.T., Sprott R.L. (1988). Biomarkers of aging. Exp. Gerontol..

[bib0015] Cawthon R.M. (2002). Telomere measurement by quantitative PCR. Nucleic Acids Res..

[bib0020] Chi Q.M., Tomita G., Inazumi K., Hayakawa T., Ido T., Kitazawa Y. (1995). Evaluation of the effect of aging on the retinal nerve fiber layer thickness using scanning laser polarimetry. J. Glaucoma.

[bib0025] Collerton J., Martin-Ruiz C., Davies K., Hilkens C.M., Isaacs J., Kolenda C., Parker C., Dunn M., Catt M., Jagger C., von Zglinicki T., Kirkwood T.B.L. (2012). Frailty and the role of inflammation, immunosenescence and cellular ageing in the very old: cross-sectional findings from the Newcastle 85+ study. Mech. Age. Dev..

[bib0030] Deeks S.G. (2009). Immune dysfunction, inflammation, and accelerated aging in patients on antiretroviral therapy. Top. HIV Med..

[bib0035] Deeks S.G. (2011). HIV infection, inflammation, immunosenescence, and aging. Annu. Rev. Med..

[bib0040] Der G., Batty G.D., Benzeval M., Deary I.J., Green M.J., McGlynn L., Mcintyre A., Robertson T., Shiels P.G. (2012). Is telomere length a biomarker for aging: cross-sectional evidence from the West of Scotland?. PLoS ONE.

[bib0045] Desquilbet L., Jacobson L.P., Fried L.P., Phair J.P., Jamieson B.D., Holloway M., Margolick J.B. (2007). HIV-1 infection is associated with an earlier occurrence of a phenotype related to frailty. J. Gerontol. A: Biol. Sci. Med. Sci..

[bib0050] Desquilbet L., Jacobson L.P., Fried L.P., Phair J.P., Jamieson B.D., Holloway M., Margolick J.B. (2011). A frailty-related phenotype before HAART initiation as an independent risk factor for AIDS or death after HAART among HIV-infected men. J. Gerontol. A: Biol. Sci. Med. Sci..

[bib0055] Desquilbet L., Margolick J.B., Fried L.P., Phair J.P., Jamieson B.D., Holloway M., Jacobson L.P. (2009). Relationship between a frailty-related phenotype and progressive deterioration of the immune system in HIV-infected men. J. Acquir. Immune Defic. Syndr..

[bib0060] Fried L.P., Tangen C.M., Walston J., Newman A.B., Hirsch C., Gottdiener J., Seeman T., Tracy R., Kop W.J., Burke G., McBurnie M.A. (2001). Frailty in older adults: evidence for a phenotype. J. Gerontol. A: Biol. Sci. Med. Sci..

[bib0065] Guaraldi G., Orlando G., Zona S., Menozzi M., Carli F., Garlassi E., Berti A., Rossi E., Roverato A., Palella F. (2011). Premature age-related comorbidities among HIV-infected persons compared with the general population. Clin. Infect. Dis..

[bib0070] Ikram M.K., De Jong F.J., Vingerling J.R., Witteman J.C.M., Hofman A., Breteler M.M.B., de Jong P.T.V.M. (2004). Are retinal arteriolar or venular diameters associated with markers for cardiovascular disorders? The Rotterdam study. Invest. Ophthalmol. Vis. Sci..

[bib0075] Jitratkosol M.H., Sattha B., Maan E.J., Gadawski I., Harrigan P.R., Forbes J.C., Alimenti A., van Schalkwyk J., Money D.M., Cote H.C. (2012). Blood mitochondrial DNA mutations in HIV-infected women and their infants exposed to HAART during pregnancy. AIDS.

[bib0080] Justice A.C., Freiberg M.S., Tracy R., Kuller L., Tate J.P., Goetz M.B., Fiellin D.A., Vanasse G.J., Butt A.A., Rodriguez-Barradas M.C., Gibert C., Oursler K.A., Deeks S.G., Bryant K. (2012). Does an index composed of clinical data reflect effects of inflammation, coagulation, and monocyte activation on mortality among those aging with HIV?. Clin. Infect. Dis..

[bib0085] Kanamori A., Escano M.F., Eno A., Nakamura M., Maeda H., Seya R., Ishibashi K., Negi A. (2003). Evaluation of the effect of aging on retinal nerve fiber layer thickness measured by optical coherence tomography. Ophthalmologica.

[bib0090] Klein R., Klein B.E.K., Knudtson M.D., Wong T.Y., Tsai M.Y. (2006). Are inflammatory factors related to retinal vessel caliber? The Beaver dam eye study. Arch. Ophthalmol..

[bib0095] Koppelstaetter C., Schratzberger G., Perco P., Hofer J., Mark W., Ollinger R., Oberbauer R., Schwarz C., Mitterbauer C., Kainz A., Karkoszka H., Wiecek A., Mayer B., Mayer G. (2008). Markers of cellular senescence in zero hour biopsies predict outcome in renal transplantation. Aging Cell.

[bib0100] Krishnamurthy J., Torrice C., Ramsey M.R., Kovalev G.I., Al-Regaiey K., Su L., Sharpless N.E. (2004). Ink4a/Arf expression is a biomarker of aging. J. Clin. Invest..

[bib0105] Leeansyah E., Cameron P.U., Solomon A., Tennakoon S., Velayudham P., Gouillou M., Spelman T., Hearps A., Fairley C., Smit de V., Pierce A.B., Armishaw J., Crowe S.M., Cooper D.A., Koelsch K.K., Liu J.P., Chuah J., Lewin S.R. (2013). Inhibition of telomerase activity by human immunodeficiency virus (HIV) nucleos(t)ide reverse transcriptase inhibitors: a potential factor contributing to HIV-associated accelerated aging. J. Infect. Dis..

[bib0110] Leung H., Wang J.J., Rochtchina E., Tan A.G., Wong T.Y., Klein R., Hubbard L.D., Mitchell P. (2003). Relationships between age, blood pressure, and retinal vessel diameters in an older population. Invest. Ophthalmol. Vis. Sci..

[bib0115] Liu Y., Johnson S.M., Fedoriw Y., Rogers A.B., Yuan H., Krishnamurthy J., Sharpless N.E. (2011). Expression of p16(INK4a) prevents cancer and promotes aging in lymphocytes. Blood.

[bib0120] Liu Y., Sanoff H.K., Cho H., Burd C.E., Torrice C., Ibrahim J.G., Thomas N.E., Sharpless N.E. (2009). Expression of p16(INK4a) in peripheral blood T-cells is a biomarker of human aging. Aging Cell.

[bib0125] Livak K.J., Schmittgen T.D. (2001). Analysis of relative gene expression data using real-time quantitative PCR and the 2(−delta delta *C*(*T*)) Method. Methods.

[bib0130] Lohse N., Hansen A.B., Pedersen G., Kronborg G., Gerstoft J., Sorensen H.T., Vaeth M., Obel N. (2007). Survival of persons with and without HIV infection in Denmark, 1995–2005. Ann. Intern. Med..

[bib0135] Lundgren J.D., Baxter J., Deeks S.G., Lane H.C. (2010). Biomark. HIV Dis..

[bib0140] Malan-Muller S., Hemmings S.M., Spies G., Kidd M., Fennema-Notestine C., Seedat S. (2013). Shorter telomere length – a potential susceptibility factor for HIV-associated neurocognitive impairments in South African woman. PLoS One.

[bib0145] Margolick J., Jacobson L., Lopez J., Li X., Martinez- Maza O., Phair J., Rinaldo C., Bream J. (2012). Frailty and circulating concentrations of proinflammatory cytokines and chemokines in HIV-infected and – uninfected men in the Multicenter AIDS Cohort Study (MACS). 3rd International Workshop on HIV & Aging, Baltimore.

[bib0150] Martin-Ruiz C., Jagger C., Kingston A., Collerton J., Catt M., Davies K., Dunn M., Hilkens C., Keavney B., Pearce S.H.S., Elzen W.P.J.d., Talbot D., Wiley L., Bond J., Mathers J.C., Eccles M.P., Robinson L., James O., Kirkwood T.B.L., von Zglinicki T. (2011). Assessment of a large panel of candidate biomarkers of ageing in the Newcastle 85+ study. Mech. Age. Develop..

[bib0155] McGlynn L.M., Stevenson K., Lamb K., Zino S., Brown M., Prina A., Kingsmore D., Shiels P.G. (2009). Cellular senescence in pretransplant renal biopsies predicts postoperative organ function. Aging Cell.

[bib0160] Mills E.J., Rammohan A., Awofeso N. (2011). Ageing faster with AIDS in Africa. Lancet.

[bib0165] Nguyen T.T., Islam F.M.A., Farouque H.M.O., Klein R., Klein B.E.K., Cotch M.F., Herrington D.M., Wong T.Y. (2010). Retinal vascular caliber and brachial flow-mediated dilation: the multi-ethnic study of atherosclerosis. Stroke.

[bib0170] Nixon D.E., Landay A.L. (2010). Biomarkers of immune dysfunction in HIV. Curr. Opin. HIV AIDS.

[bib0175] Pathai S., Gilbert C., Weiss H.A., Cook C., Wood R., Bekker L.G., Lawn S.D. (2013). Frailty in HIV-infected adults in South Africa. J. Acquir. Immune Defic. Syndr..

[bib0180] Pathai S., Gilbert C.E., Lawn S.D., Weiss H.A., Peto T., Cook C., Wong T.Y., Shiels P.G. (2013). Assessment of candidate ocular biomarkers of ageing in a South African adult population: relationship with chronological age and systemic biomarkers. Mech. Age. Dev..

[bib0185] Pathai S., Lawn S.D., Shiels P.G., Weiss H.A., Cook C., Wood R., Gilbert C.E. (2013). Corneal endothelial cells provide evidence of accelerated cellular senescence associated with HIV infection: a case-control study. PLoS One.

[bib0190] Pathai S., Lawn S.D., Weiss H.A., Cook C., Bekker L.G., Gilbert C. (2013). Increased ocular lens density in HIV-infected individuals with low nadir CD4 counts in South Africa: evidence of accelerated aging. J. Acquir. Immune Defic. Syndr..

[bib0195] Pathai S., Shiels P.G., Lawn S.D., Cook C., Gilbert C. (2013). The eye as a model of ageing in translational research – Molecular epigenetic and clinical aspects. Age. Res. Rev..

[bib0200] Pathai S., Weiss H.A., Lawn S.D., Peto T., D’Costa L.M., Cook C., Wong T.Y., Gilbert C.E. (2012). Retinal arterioles narrow with increasing duration of anti-retroviral therapy in HIV infection: a novel estimator of vascular risk in HIV?. PLoS ONE.

[bib0205] Payne B.A.I., Wilson I.J., Hateley C.A., Horvath R., Santibanez-Koref M., Samuels D.C., Price D.A., Chinnery P.F. (2011). Mitochondrial aging is accelerated by anti-retroviral therapy through the clonal expansion of mtDNA mutations. Nat. Pub. Group.

[bib0210] Saretzki G., Von Zglinicki T. (2002). Replicative aging, telomeres, and oxidative stress. Ann. N. Y. Acad. Sci..

[bib0215] Shay J.W., Wright W.E. (2000). Hayflick, his limit, and cellular ageing. Nat. Rev. Mol. Cell. Biol..

[bib0220] Shiels P.G. (2010). Improving precision in investigating aging: why telomeres can cause problems. J. Gerontol. A: Biol. Sci. Med. Sci..

[bib0225] Simm A., Nass N., Bartling B., Hofmann B., Silber R.-E., Navarrete Santos A. (2008). Potential biomarkers of ageing. Biol. Chem..

[bib0230] Simm A., Nass N., Bartling B., Hofmann B., Silber R.E., Navarrete Santos A. (2008). Potential biomarkers of ageing. Biol. Chem..

[bib0235] Smith R.L., de Boer R., Brul S., Budovskaya Y., van Spek H. (2012). Premature and accelerated aging: HIV or HAART?. Front. Genet..

[bib0240] Triant V.A. (2012). HIV infection and coronary heart disease: an intersection of epidemics. J. Infect. Dis..

[bib0245] von Zglinicki T. (2002). Oxidative stress shortens telomeres. Trends Biochem. Sci..

[bib0250] von Zglinicki T. (2012). Will your telomeres tell your future?. BMJ.

[bib0255] Wong T.Y., Islam F.M.A., Klein R., Klein B.E.K., Cotch M.F., Castro C., Sharrett A.R., Shahar E. (2006). Retinal vascular caliber, cardiovascular risk factors, and inflammation: the multi-ethnic study of atherosclerosis (MESA). Invest. Ophthalmol. Vis. Sci..

[bib0260] Wong T.Y., Klein R., Klein B.E., Meuer S.M., Hubbard L.D. (2003). Retinal vessel diameters and their associations with age and blood pressure. Invest. Ophthalmol. Vis. Sci..

[bib0265] Wong T.Y., Klein R., Sharrett A.R., Duncan B.B., Couper D.J., Tielsch J.M., Klein B.E.K., Hubbard L.D. (2002). Retinal arteriolar narrowing and risk of coronary heart disease in men and women: the atherosclerosis risk in communities study. JAMA.

[bib0270] Wong T.Y., Knudtson M.D., Klein B.E.K., Klein R., Hubbard L.D. (2005). Estrogen replacement therapy and retinal vascular caliber. Ophthalmology.

[bib0275] Wong T.Y., Knudtson M.D., Klein R., Klein B.E.K., Meuer S.M., Hubbard L.D. (2004). Computer-assisted measurement of retinal vessel diameters in the Beaver dam eye study: methodology, correlation between eyes, and effect of refractive errors. Ophthalmology.

[bib0280] Yamaguchi T., Takayama Y., Saito M., Ishikawa F., Saneyoshi M. (2001). Telomerase-inhibitory effects of the triphosphate derivatives of some biologically active nucleosides. Nucleic Acids Res..

