# Supplementary material for: Ocular parameters of biological ageing in HIV-infected individuals in South Africa: Relationship with chronological age and systemic biomarkers of ageing
Source: Mech Ageing Dev. 2013 Sep;134(9):400–6. doi: 10.1016/j.mad.2013.08.002 (PMC3818088; doi:10.1016/j.mad.2013.08.002)
Supplement: Supplementary file 1 [file mmc1.docx]

**Table S1: Characteristics of study participants, n=216**

| **Variable** | **Male (N=54)**  **N (%)** | **Female (N=162)**  **N (%)** | **P** |
| --- | --- | --- | --- |
| Age (mean ± SE) | 41.5±0.9 | 41.2±0.6 | 0.82 |
| Median age, IQR | 40 (37-46) | 40 (35-47) | 0.52 |
| Age, years by group  30-39  40-49  >50 | 25 (46.3)  22 (40.7)  7 (13.0) | 80 (49.4)  52 (30.1)  30 (18.5) | 0.43 |
| Education  Did not complete high school  Completed high school | 9 (16.7)  45 (83.3) | 16 (9.9)  146 (90.1) | 0.18 |
| Income  <ZAR1000/month  >ZAR1000/month | 32 (59.3)  22 (40.7) | 89 (54.9)  73 (45.1) | 0.58 |
| Location of work  Outdoors or grant-holder  Indoors | 38 (70.4)  16 (29.6) | 98(60.5)  64(39.5) | 0.19 |
| Housing  Formal  Informal | 24 (44.4)  30 (55.6) | 88 (54.3)  74 (45.7) | 0.21 |
| Smoking status  Non-smoker  Smoker; <10 years  Smoker >10 years | 32 (59.3)  5 (9.3)  17(31.4) | 152 (93.8)  5 (3.1)  5 (3.1) | <0.0001 |
| Alcohol  Nil  <1L/week  >1L/week | 25 (46.3)  18 (33.3)  11 (20.4) | 126 (77.8)  23 (14.2)  13 (8.0) | <0.0001 |
| Hypertension  No  Yes | 42 (77.8)  12 (22.2) | 111 (68.5)  51 (31.5) | 0.20 |
| BMI  <20  20-24.9  25.0-29.9  >30 | 12 (22.2)  29 (53.7)  10 (18.5)  3 (5.6) | 5 (3.1)  31(19.1)  56 (34.6)  70 (42.2) | <0.0001 |
| Tuberculosis status  No history  Previous history  Current | 8 (14.8)  43 (79.6)  3 (5.6) | 52 (32.1)  109 (67.3)  1 (0.6) | 0.005 |
| Current CD4 count, median (IQR) | 410  (270-610) | 481  (345-607) | 0.04 |
| Nadir CD4 count, median (IQR) | 135  (81-166) | 122  (70-174) | 0.69 |
| Viral load  <50 copies/ml  >50 copies/ml | 49 (90.7)  5 (9.3) | 154 (95.0)  8 (5.0) | 0.25 |
| Duration of ART*, median (IQR) | 55  (41-72) | 60  (33-76) | 0.96 |

*ART; anti-retroviral treatment

**Table S2: Median values of candidate biomarkers by gender and age group**

*P-value for difference between genders*

|  |  | **Men** | | | **Women** | | | **P-value** |
| --- | --- | --- | --- | --- | --- | --- | --- | --- |
| **Measure** | **Age group, years** | **N** | **Median** | **IQR** | **N** | **Median** | **IQR** |  |
| **Systemic biomarkers** |  |  |  |  |  |  |  |  |
| Telomere length  Rel T/S | Overall | 48 | 0.96 | 0.78-1.09 | 156 | 0.92 | 0.78-0.92 | 0.51 |
|  | 30-39 | 23 | 1.00 | 0.80-1.13 | 78 | 0.97 | 0.79-1.10 |  |
|  | 40-49 | 20 | 0.94 | 0.81-1.07 | 49 | 0.91 | 0.78-1.11 |  |
|  | >50 | 5 | 0.74 | 0.64-1.27 | 29 | 0.82 | 0.74-1.00 |  |
| CDKN2A expression | Overall | 47 | 0.46 | 0.24-0.72 | 152 | 0.50 | 0.29-0.70 | 0.48 |
|  | 30-39 | 22 | 0.34 | 0.21-0.65 | 77 | 0.50 | 0.27-0.70 |  |
|  | 40-49 | 20 | 0.50 | 0.25-0.81 | 46 | 0.47 | 0.31-0.66 |  |
|  | >50 | 5 | 0.68 | 0.41-0.81 | 29 | 0.56 | 0.42-0.74 |  |
| **Lens density**  **Scale 0-100** |  |  |  |  |  |  |  |  |
| Linear | Overall | 52 | 9.8 | 9.2-10.5 | 157 | 9.8 | 9.3-10.5 | 0.42 |
|  | 30-39 | 24 | 9.2 | 8.9-9.7 | 77 | 9.4 | 9.0-9.6 |  |
|  | 40-49 | 22 | 10 | 9.8-10.6 | 50 | 10.3 | 9.8-10.9 |  |
|  | >50 | 6 | 11.0 | 10.5-12.1 | 30 | 11.5 | 11.0-12.0 |  |
| Peak | Overall | 52 | 17.9 | 14.9-19.5 | 157 | 17.9 | 15.6-20.1 | 0.57 |
|  | 30-39 | 24 | 18.0 | 15.4-18.8 | 77 | 16 | 14.7-18.1 |  |
|  | 40-49 | 22 | 16.8 | 14.8-19.1 | 50 | 18.5 | 16.1-21.1 |  |
|  | >50 | 6 | 21.6 | 15.8-25.0 | 30 | 20.5 | 18.8-24.7 |  |
| 3-D average | Overall | 52 | 9.7 | 9.1-10.3 | 157 | 9.8 | 9.1-10.8 | 0.33 |
|  | 30-39 | 24 | 9.1 | 8.8-9.7 | 77 | 9.1 | 8.9-9.5 |  |
|  | 40-49 | 22 | 10 | 9.5-10.3 | 50 | 10.3 | 9.8-10.8 |  |
|  | >50 | 6 | 11.2 | 10.5-12.5 | 30 | 11.7 | 11.0-12.4 |  |
| **Retinal vessel calibre μm** |  |  |  |  |  |  |  |  |
| CRAE | Overall | 50 | 161.7 | 151.5-170.6 | 157 | 164.8 | 152.5-173.4 | 0.36 |
|  | 30-39 | 22 | 160.7 | 151.5-167.6 | 77 | 167.1 | 155.5-176.5 |  |
|  | 40-49 | 22 | 164.1 | 158.7-172.4 | 51 | 161.7 | 152.3-171.9 |  |
|  | >50 | 6 | 139.3 | 122.1-170.3 | 29 | 162.4 | 144.8-169.4 |  |
| CRVE | Overall | 50 | 262.2 | 250.6-273.9 | 157 | 266.2 | 255.7-279.9 | 0.15 |
|  | 30-39 | 22 | 263.8 | 258.3-274.4 | 77 | 269.9 | 259.8-282.8 |  |
|  | 40-49 | 22 | 260.9 | 246.2-272.9 | 51 | 263.3 | 251.9-276.1 |  |
|  | >50 | 6 | 260.7 | 238.1-277.4 | 29 | 263.7 | 254.2-271.3 |  |
| **Endothelial cell parameters** |  |  |  |  |  |  |  |  |
| ECD | Overall | 48 | 2598 | 2337-2730 | 150 | 2690 | 2463-2858 | 0.03 |
|  | 30-39 | 23 | 2671 | 2514-2850 | 74 | 2720 | 2554-2880 |  |
|  | 40-49 | 20 | 2466 | 2125-2762 | 49 | 2665 | 2400-2816 |  |
|  | >50 | 5 | 2402 | 2122-2590 | 27 | 2566 | 2382-2729 |  |
| CV | Overall | 48 | 36 | 34-38 | 150 | 36 | 33-39 | 0.84 |
|  | 30-39 | 23 | 36 | 34-39 | 74 | 35 | 32-37 |  |
|  | 40-49 | 20 | 36 | 33-38 | 49 | 37 | 33-39 |  |
|  | >50 | 5 | 36 | 33-41 | 27 | 38 | 34-41 |  |
| Ex | Overall | 48 | 49 | 45-54 | 50 | 50 | 45-54 | 0.91 |
|  | 30-39 | 23 | 49 | 44-54 | 74 | 50 | 45-53 |  |
|  | 40-49 | 20 | 50 | 46-54 | 49 | 50 | 46-55 |  |
|  | >50 | 5 | 46 | 38-53 | 27 | 48 | 42-51 |  |
| **RNFL thickness, μm** |  |  |  |  |  |  |  |  |
| Average | Overall | 45 | 110 | 101-116 | 143 | 112 | 102-118 | 0.47 |
|  | 30-39 | 21 | 115 | 106-117 | 74 | 114 | 107-122 |  |
|  | 40-49 | 19 | 107 | 98-115 | 42 | 114 | 100-117 |  |
|  | >50 | 5 | 102 | 93-113 | 27 | 102 | 89-113 |  |
| Superior | Overall | 50 | 132 | 124-145 | 147 | 137 | 121-151 | 0.22 |
|  | 30-39 | 23 | 133 | 125-145 | 74 | 142 | 128-157 |  |
|  | 40-49 | 21 | 132 | 125-145 | 46 | 131 | 120-146 |  |
|  | >50 | 6 | 119 | 100-130 | 27 | 126 | 109-142 |  |
| Inferior | Overall | 50 | 133 | 121-144 | 149 | 141 | 124-152 | 0.18 |
|  | 30-39 | 23 | 141 | 126-156 | 75 | 144 | 129-154 |  |
|  | 40-49 | 21 | 129 | 123-142 | 46 | 142 | 118-149 |  |
|  | >50 | 6 | 119 | 96-134 | 28 | 124 | 110-141 |  |
| Nasal | Overall | 50 | 92 | 76-104 | 147 | 94 | 76-103 | 0.92 |
|  | 30-39 | 23 | 89 | 76-103 | 74 | 93 | 78-101 |  |
|  | 40-49 | 21 | 94 | 77-107 | 46 | 94 | 75-110 |  |
|  | >50 | 6 | 87 | 52-134 | 27 | 91 | 61-99 |  |
| Temporal | Overall | 50 | 73 | 64-81 | 148 | 73 | 63-84 | 0.63 |
|  | 30-39 | 23 | 74 | 65-83 | 74 | 78 | 67-88 |  |
|  | 40-49 | 21 | 71 | 64-77 | 46 | 69 | 57-78 |  |
|  | >50 | 6 | 65 | 49-82 | 28 | 66 | 60-73 |  |
| **Frailty status** |  | **N** | **%** |  | **N** | **%** |  | **P-value** |
| Non-frail | Overall | 20 | 37.0 |  | 50 | 30.8 |  | 0.56 |
|  | 30-39 | 10 | 40.0 |  | 32 | 40.0 |  |  |
|  | 40-49 | 9 | 40.9 |  | 16 | 30.8 |  |  |
|  | >50 | 1 | 14.3 |  | 2 | 6.7 |  |  |
| Pre-frail | Overall | 27 | 50.0 |  | 80 | 49.4 |  | 0.68 |
|  | 30-39 | 12 | 48.0 |  | 38 | 47.5 |  |  |
|  | 40-49 | 9 | 40.9 |  | 30 | 57.7 |  |  |
|  | >50 | 6 | 85.7 |  | 12 | 40.0 |  |  |
| Frail | Overall | 7 | 13.0 |  | 32 | 19.8 |  | 0.03 |
|  | 30-39 | 3 | 12.0 |  | 10 | 12.5 |  |  |
|  | 40-49 | 4 | 18.2 |  | 6 | 11.5 |  |  |
|  | >50 | 0 | 0 |  | 16 | 53.3 |  |  |

**Table S3: Regression coefficients of biomarkers with chronological age in years**

| **Biomarker** | **N** | **Coefficient** | **R-squared** | **P-value** |
| --- | --- | --- | --- | --- |
| Telomere length | 204 | -0.002 | 0.02 | 0.03 |
| CDKN2A | 199 | 0.003 | 0.007 | 0.25 |
|  |  |  |  |  |
| *Lens density* |  |  |  |  |
| Linear | 209 | 0.11 | 0.63 | <0.0001 |
| Peak | 209 | 0.23 | 0.18 | <0.0001 |
| Average 3D | 246 | 0.13 | 0.71 | <0.0001 |
|  |  |  |  |  |
| *Retinal vessel calibre* |  |  |  |  |
| Arteriolar diameter | 207 | -0.40 | 0.03 | 0.01 |
| Venular diameter | 207 | -0.58 | 0.06 | 0.001 |
|  |  |  |  |  |
| *Endothelial cell parameters*** |  |  |  |  |
| ECD | 198 | -9.91 | 0.06 | <0.0001 |
| CV | 198 | 0.13 | 0.06 | <0.0001 |
| Ex | 198 | -0.12 | 0.01 | 0.12 |
|  |  |  |  |  |
| *Retinal nerve fibre layer thickness* |  |  |  |  |
| Average | 188 | -0.60 | 0.14 | <0.0001 |
| Superior | 197 | -0.89 | 0.10 | <0.0001 |
| Inferior | 199 | -0.95 | 0.11 | <0.0001 |
| Nasal | 197 | -0.20 | 0.005 | 0.30 |
| Temporal | 198 | -0.46 | 0.06 | <0.0001 |

Linear regression models using age in years as a continuous variable in years

**ECD: endothelial cell density - lowest quartile denotes aged phenotype

CV: coefficient of variation – i.e. difference in cell shape; highest quartile denotes aged phenotype

Ex: Hexagonality index – i.e. proportion of cells that are hexagonal; lowest quartile denotes aged phenotype
