# Supplementary figures and images for: Ocular parameters of biological ageing in HIV-infected individuals in South Africa: Relationship with chronological age and systemic biomarkers of ageing
Source: Mech Ageing Dev. 2013 Sep;134(9):400–6. doi: 10.1016/j.mad.2013.08.002 (PMC3818088; doi:10.1016/j.mad.2013.08.002)

**Supplementary Figures – See main manuscript for captions**

1a:


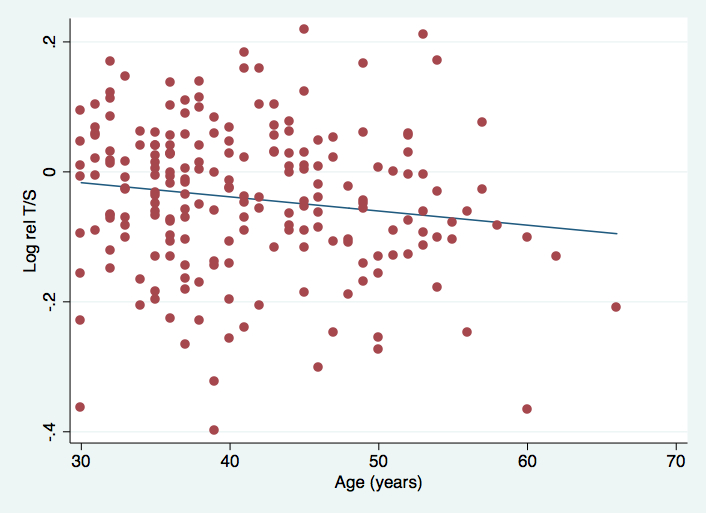


1b:


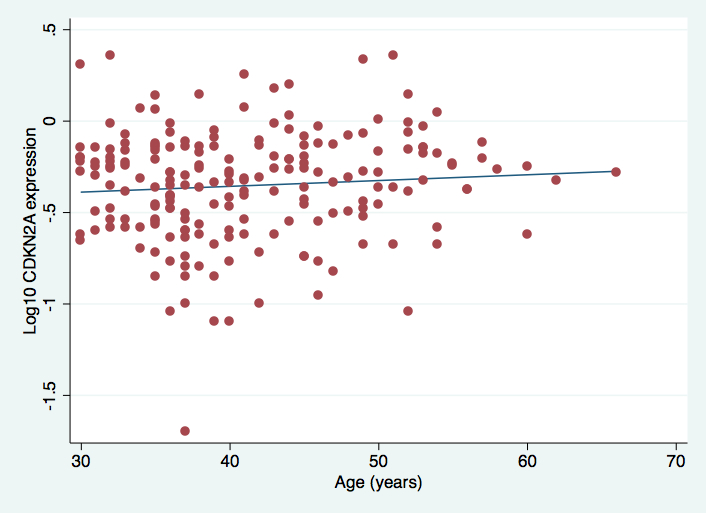


1c:


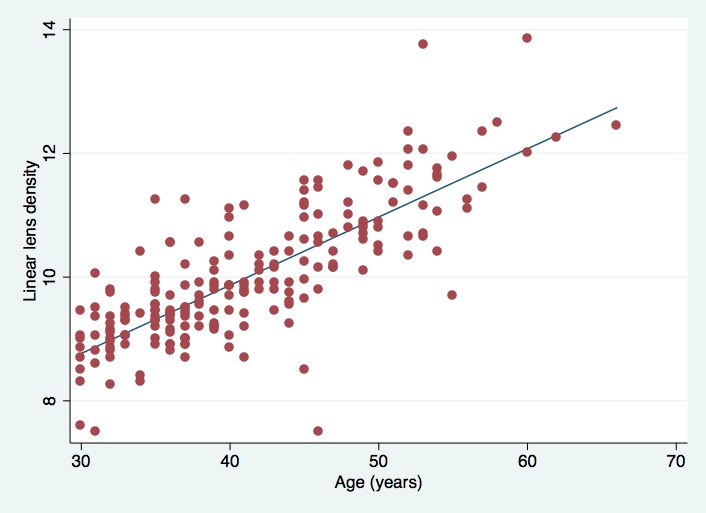


1d:


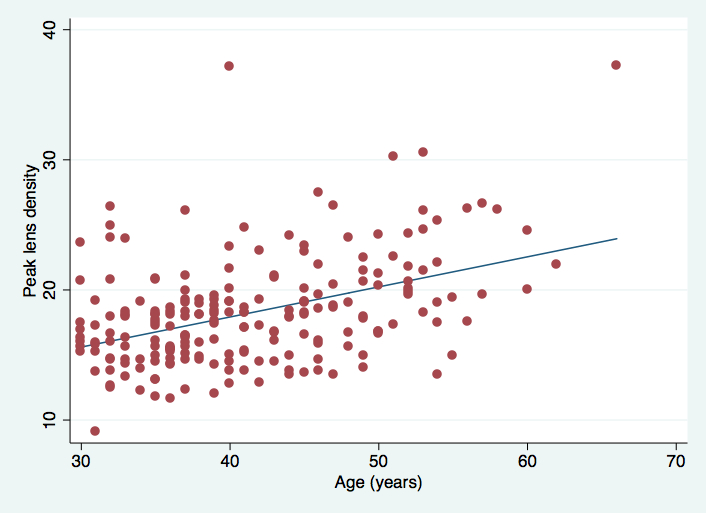


1e:


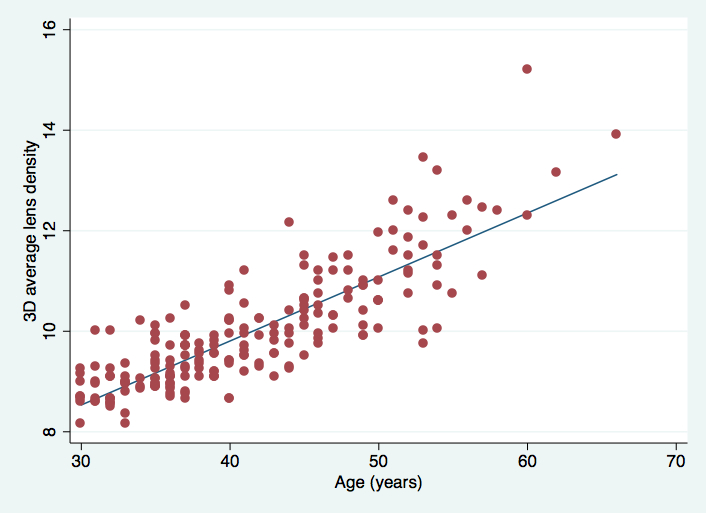


1f:


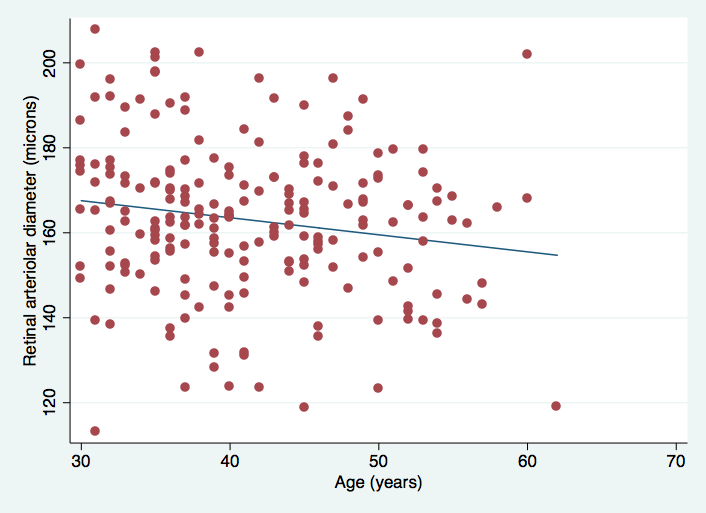


1g:


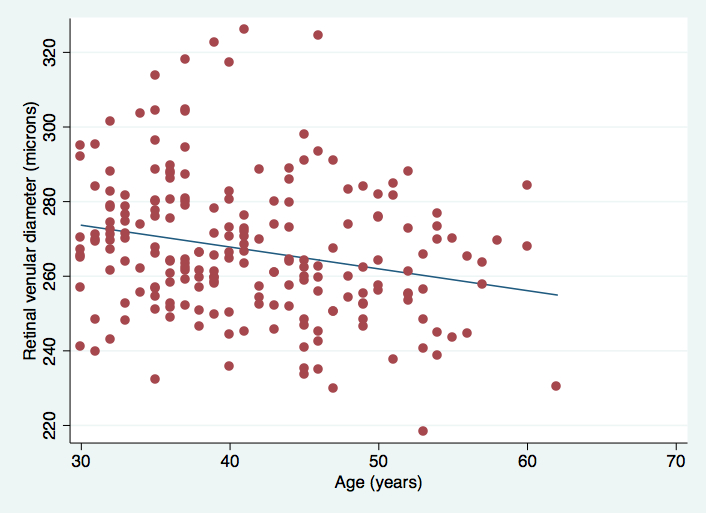


1h:


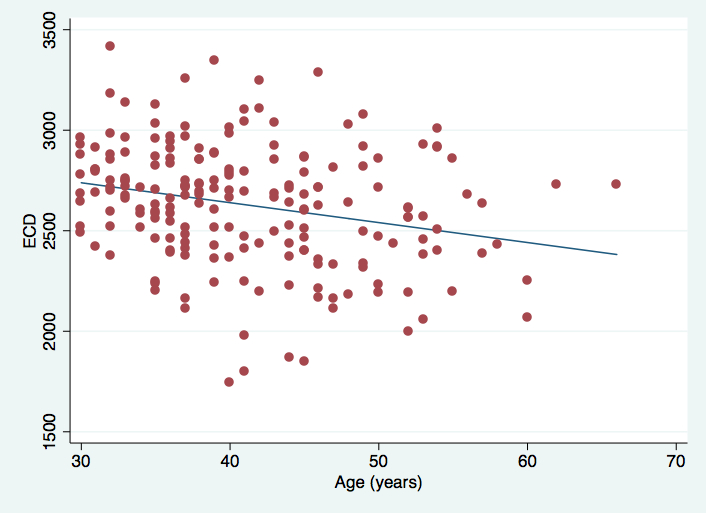


1i:


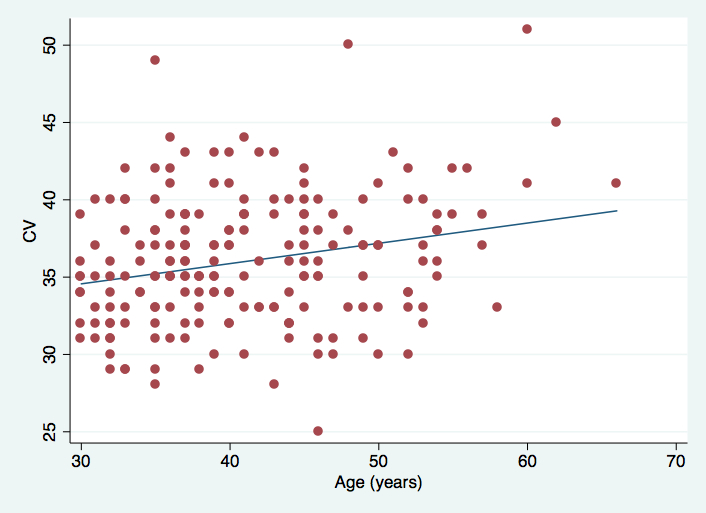


1j:


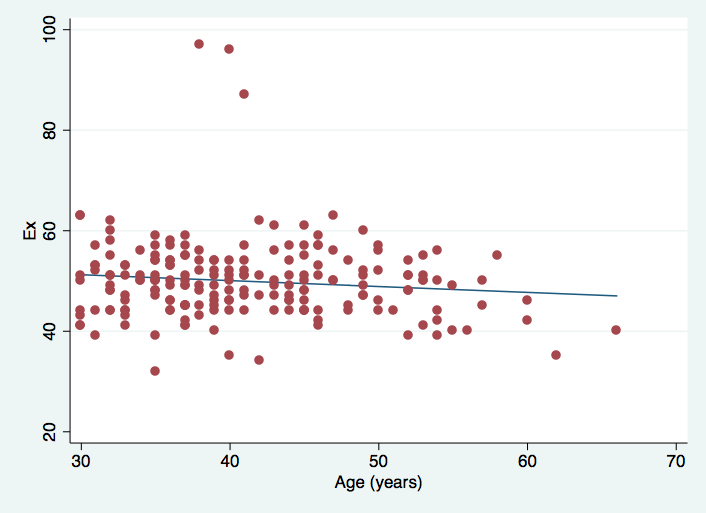


1k:


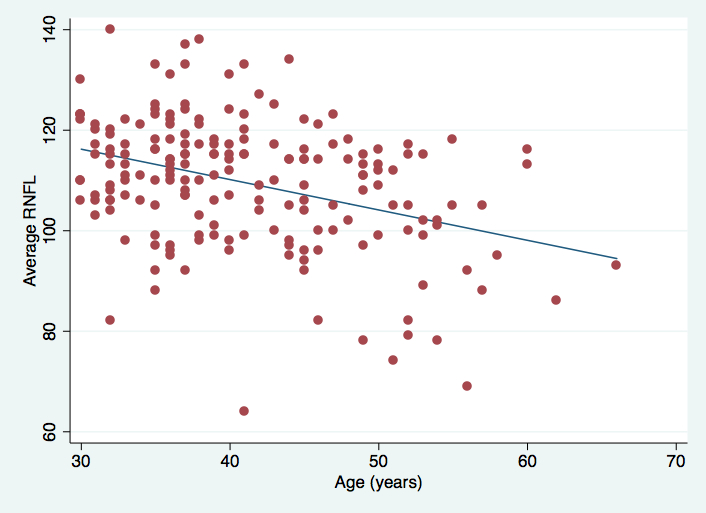


1l:


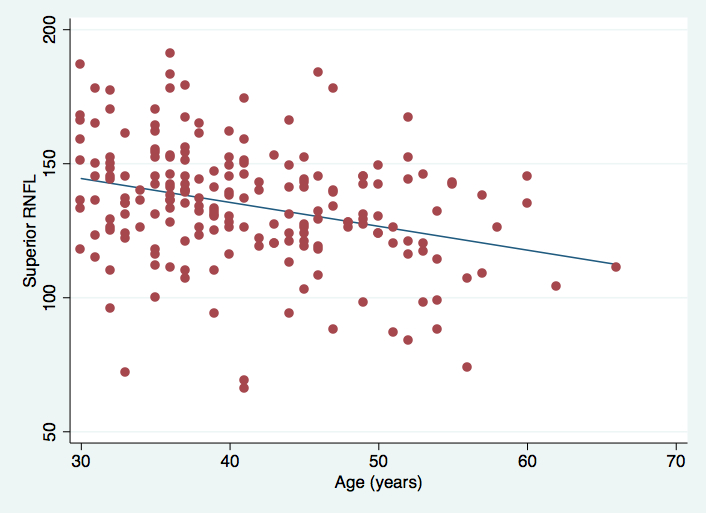


1m:


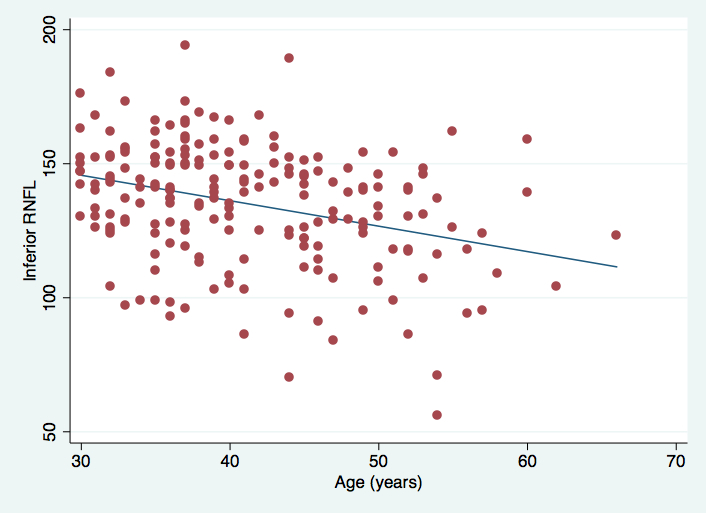


1n:


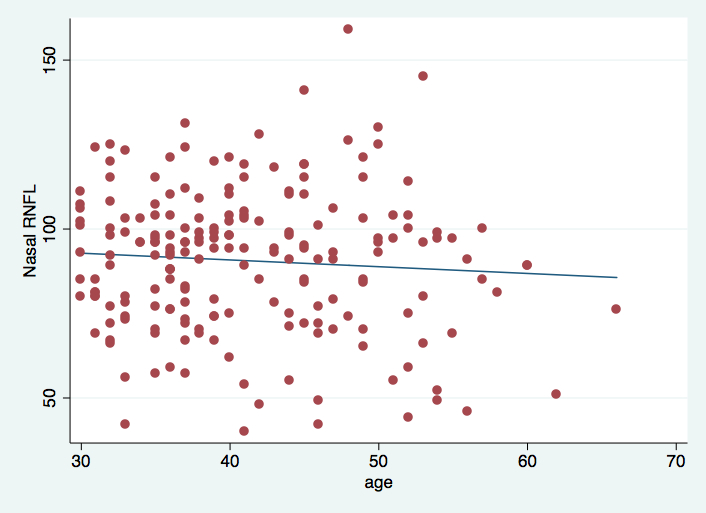


1o:


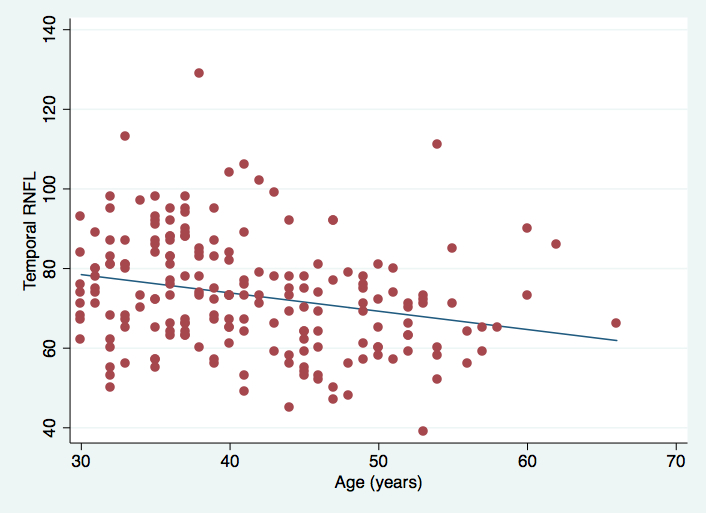

Supplement: Supplementary file 2 [file mmc2.docx]
